# Stakeholders' Perceptions of Integrated Community Case Management by Community Health Workers: A Post-Intervention Qualitative Study

**DOI:** 10.1371/journal.pone.0098610

**Published:** 2014-06-13

**Authors:** Denise L. Buchner, Jennifer L. Brenner, Jerome Kabakyenga, Kyomuhangi Teddy, Samuel Maling, Celestine Barigye, Alberto Nettel-Aguirre, Nalini Singhal

**Affiliations:** 1 Faculty of Medicine, University of Calgary, Calgary, Alberta, Canada; 2 Faculty of Medicine, Mbrara University of Science and Technology, Mbrara District, Mbrara, Uganda; 3 Healthy Child Uganda, Mbrara District, Mbrara, Uganda; 4 Ministry of Health, Government of Uganda, Kampala, Uganda; Centers for Disease Control and Prevention, United States of America

## Abstract

**Background:**

Integrated community case management (iCCM) involves delivery of simple medicines to children with pneumonia, diarrhea and/or malaria by community health workers (CHWs). Between 2010 and 2012, an iCCM intervention trial was implemented by Healthy Child Uganda. This study used qualitative tools to assess whether project stakeholders perceived that iCCM improved access to care for children under five years of age.

**Methods:**

The intervention involved training and equipping 196 CHWs in 98 study villages in one sub-county in Uganda in iCCM. During the eight-month intervention, CHWs assessed sick children, provided antimalarials (coartem) for fever, antibiotics (amoxicillin) for cough and fast breathing, oral rehydration salts/zinc for diarrhea, and referred very sick children to health facilities. In order to examine community perceptions and acceptability of iCCM, post-intervention focus groups and key respondent interviews involving caregivers, health workers, CHWs and local leaders were carried out by experienced facilitators using semi-structured interview guides. Data were analyzed using thematic analysis techniques.

**Results:**

Respondents reported increased access to health care for children as a result of iCCM. Access was reportedly closer to home, available more hours in a day, and the availability of CHWs was perceived as more reliable. CHW care was reported to be trustworthy and caring. Families reported saving money especially due to reduced transportation costs, and less time away from home. Respondents also perceived better health outcomes. Linkages between health facilities and communities were reportedly improved by the iCCM intervention due to the presence of trained CHWs in the community.

**Conclusions:**

iCCM delivered by CHWs may improve access to health care and is acceptable to families. Policymakers should continue to seek opportunities to implement and support iCCM, particularly in remote communities where there are health worker shortages.

## Introduction

Globally, many children still die from preventable and easily treatable infections, including malaria, pneumonia, and diarrhea. In rural Sub-Saharan Africa, the situation is aggravated by long distances to clinics, limited formal health facilities, and few or no trained health professionals available at the community level.

In Uganda, under-five mortality remains high at 199/1000 live births [1]. There is growing recognition that in low-resource settings with shortages of trained health care staff, community-based health programs can reduce mortality and morbidity of young children, and support health service delivery [1], [2], [3].

‘Task shifting’ involves the redistribution of health service provision to less specialized health workers in order to bring essential health services closer to populations with limited or no access to essential public health services [4]. Integrated Community case management (iCCM) involves using lay individuals trained as community health workers (CHWs) to provide curative treatment within their communities. Since 2004, when UNICEF and the World Health Organization issued a joint statement on iCCM[5], iCCM programs have demonstrated success in treating childhood illnesses including fever/malaria [6], pneumonia [7], and diarrhea [8]. iCCM has been proposed over the past decade as a child health strategy to reduce under-five mortality and as means of improving progress toward Millennium Development Goal 4 (MDG) [9]. Few qualitative studies have examined community perceptions of iCCM [10], [11].

Access to health care in low-resource countries is often discussed in terms of ‘barriers’ [12]. A 2010 systematic review of access to health services for children under-five years in sub-Sarahan Africa [13], describes ‘traditional’ and “other” measures of health care access. One traditional ‘access’ measure is distance to nearest health facility. Numerous studies have demonstrated that families who live more than five kilometers from a health facility are less likely to access services at the facility, and more likely to seek health care from informal providers closer to home. One recent Ugandan study found that a distance exceeding three kilometers created a barrier to health facility access [14]. Another traditional access measure is cost, which is often described as user fees charged by health facilities, although other often overlooked costs like transportation, prescription medicine, accommodation and food should also be considered [14], [15]. Other frequently overlooked barriers to health care access include lack of social support, health knowledge, decision-making power, availability of time, and autonomy of primary caregiver [13], [16]. Factors enhancing health care ‘access’ can include: the positive manner of health care provision, the availability drugs that are perceived to be helpful, and the timeliness of care [17].

In 2010, iCCM was formally introduced as a key component of the Ugandan national health strategy[18]. Between 2010 and 2012, a Ugandan-Canadian partnership called Healthy Child Uganda (HCU) implemented an iCCM intervention in rural southwestern Uganda as a pilot study to evaluate the newly proposed iCCM program [19]. A post-intervention study of the HCU iCCM intervention reported significant increases in children who accessed appropriate care for common childhood illnesses in intervention areas. Of the 6276 sick children reported by CHWs, 93% received treatments consistent with iCCM algorithms. Moreover, in intervention communities, mothers reported a 24% increase of children receiving antimalarial medicine (from any source) for fever and a 14% increase for children receiving rehydration salts and zinc for diarrhea (compared to 4% and 1% increase respectively in control communities). Post intervention, 64% of children in intervention areas received antibiotics for presumed pneumonia compared to only 28% in control communities [20] [21]. This paper describes a complementary post-intervention qualitative study to assess stakeholder perceptions of the HCU iCCM intervention. Overall, stakeholders reported positive perceptions of the HCU iCCM intervention and requested that the project continue.

## Methods

### Ethics

Ethical approval for this study was obtained from the Conjoint Health Research Ethics Board at the University of Calgary (E-24432), the Clinical Research Ethics Board at the University of British Columbia (HII-00947), and Institutional Review Committees at the Mbarara University of Science and Technology (March 11, 2011). The study was registered at clinicaltrials.gov (NCT02046018).

### Setting and Intervention

This study was conducted in Kyabugimbi sub-county (population 37,200), where the iCCM intervention occurred. Located in Bushenyi District in southwestern Uganda, Kyabugimbi sub-county is a rural area with steep, mountainous terrain and few roads, mostly unpaved. Most families in the area rely on subsistence farming for survival and few have access to electricity, running water or modern sanitation systems. Access to government health centres is limited, and health services at existing facilities are challenged by staffing, equipment shortages and limited infrastructure.

In 2010, 198 CHWs were selected and trained in 98 villages in the intervention sub-county. Selection of CHW's occurred through a nomination process (show of hands) at community meetings. Community members were asked to select CHW's based on the individual's age (18+years), perceived trustworthiness, demonstrated willingness to volunteer, years of residency in the community, and the ability to be available in the community. Level of education was not a factor for CHW selection. Each village selected 2 to 5 CHWs depending on the population size of the community; these individuals attended a five-day basic health promotion course, conducted by local health centre staff and in accordance with Ministry of Health guidelines, which followed a standard curriculum set by the Government of Uganda [18]. All CHWs provided health education and promoted healthy practices within their respective communities. In April 2012, two CHWs from each village were selected (by community nomination) to receive additional iCCM training. Following five days of iCCM training, these CHWs began assessing and providing pre-packaged drugs to treat uncomplicated illnesses—antimalarials (coartem) for fever, antibiotics (amoxicillin) for presumed pneumonia, and oral rehydration salts/zinc for diarrhea—to sick children under five years of age in their communities.

About half of the CHWs trained in iCCM were also enrolled in an enhanced study arm and were provided with mobile phones to supplement iCCM provision. CHWs selected for the cell phone arm of the study were nominated into the role at community meetings using the same guidelines employed for the selection of non-cell phone CHWs. CHWs with study-supplied phones used a mobile application to report the age of the child and his/her symptoms; the application then guided the CHW through a treatment algorithm that suggested appropriate treatment. Data entered into study-supplied phones was transmitted immediately to a database located at a local health facility; health centre staff reviewed the database daily.

All CHWs were supervised and supported by local health centre staff who reviewed CHW reports, either paper or cell phone generated, met monthly with CHW's to discuss the program and address problems, and communicated with CHWs regarding referrals or follow-up of patients discharged from hospital. Health centre staff were also available to consult with CHW's regarding complicated cases as needed.

### Data Collection and Analysis

This qualitative study was part of a larger research effort, which included a quantitative component with household surveys in intervention and control communities, as well as a review of operational data. Further details on the larger study are published elsewhere [20]

Focus group discussions (FGDs) and key informant interviews (KIIs) were designed using a phenomenology framework [22] and were conducted post-intervention with key stakeholders who were involved with the HCU iCCM project in intervention communities. FGDs and KIIs tools were semi-structured, developed in English, translated into the local language (Runyankore) and then back-translated into English to ensure accuracy of translation. Interview tools included open-ended questions that addressed stakeholders impressions of the iCCM by CHW intervention; access to care; perceived satisfaction and quality of care; referral; patient encounters; supervision; and drug supply. A different interview tool was created for each stakeholder group. Sample FGD/KII questions are presented in Table 1. FGDs commenced directly after completion of household surveys, which permitted preliminary results from the household survey to inform the development of questions for the FGDs and KIIs.

**Table 1:** Sample of Questions from Focus Group Discussions and Key Informant Interviews.

Sample Questions for Community Health Workers (CHWs)
1 In what areas of your work as a VHT do you feel most confident?
2 In your work as a VHT, what are some of the challenges you have faced?
3 Can you describe your connection with health centres?
4 Do you feel qualified to distribute drugs to young children in your community?
Sample Questions for Caregivers
1 From you experiences, do you think VHTs have enough knowledge and skills to determine danger signs in children and also administer drugs to young children?
2 Do you think that the VHT program in your community is improving the health of women and young children?
Sample Questions for Health Workers
1 How do you think VHTs feel about their expanded role as health providers in communities?
2 Can you tell us what worked well and what the challenges were with regards to referrals made by VHTs?
3 We are also interested to know about supervision of VHTs. From your experience how are VHTs being supervised? =
4 Do you think the iCCM and/or cell phone project improved health outcomes in Kyabugimbi sub-district?
Sample Questions for Local Leaders
1 What is your opinion about the ability of VHTs to deliver basic drugs and health interventions for women and children in rural areas?
2 From your perspective, what do you think community members think about the acceptability of VHTs distributing drugs to young children?
3 If were were to continue with the [iCCM/cell phone] project what could we do better?

Note: In Uganda CHWs are called Village Health Teams (VHTs) and are referred to as VHTs in these questions.

FGDs and KIIs were conducted in December 2012, following eight months of iCCM intervention. Participants were caregivers of children under five years, CHWs, health centre staff, local leaders and district government officials. A caregiver was defined as any adult who looks after the day-to-day needs of the child (provides food, shelter, safety). KII participants were purposely selected due to their involvement in or exposure to the intervention. FGD CHW participants were randomly selected using Rand's random digit sampling strategy [23] based on CHW lists. Caregivers (M/F) were eligible for participation in a FGD if they had a child under five years of age and had consulted an ICCM CHW for a sick child during the previous six months. Eligible caregivers were identified by CHWs. Due to small numbers and close proximity, all local leaders (12) and health centre staff (13) involved in the study were invited to FGDs. Government officials (5) were selected for a KII due to their involvement in or knowledge about the program. The sampling method for FGDs is detailed further in Table 2.

**Table 2:** Sampling Strategy for Focus Group Discussions.

Caregivers (6 FGDs)	1 Six communities randomly selected
	2 Male and Female caregivers identified by CHWs for intervention FGDs (three male and three female FDGs)
	3 Criteria for selection was that the caregiver should 1) live in the village and 2) a child in the family was taken to a CHW for an illness episode at any time in the last six months.
CHWs (6 FDGs)	1 Six communities randomly selected
	2 A random sample of eight CHWs from CHW database were invited to the FDGs.
Health Centre Staff (1 FGD)	All health centre staff (13) who were also iCCM trainers were invited to the FGD.
Local Leaders (2 FGDs)	1 Two communities randomly selected.
	2 All local leaders from the selected communities were invited to the FGDs.

Focus Group Discussions were held in nine intervention parishes in Kyabugimbi Sub-county in November and December 2012.

Experienced facilitators (2 males, 1 female) from Mbrara University of Science and Technology facilitated the FGD's and KIIs. None of these facilitators were previously involved in the project implementation or had a vested interest in the outcomes of the study. FGDs with caregivers, CHWs and local leaders were conducted in communities, within walking distance of the homes of participants, at local churches or community gathering places. Locations provided privacy and no non-participants were present. The FGD with health workers was held at a health center in a private meeting room. KIIs were held at offices or in private residences and in privacy. All FGDs included both male and female participants together except the caregiver groups, which were separated by gender. All FGDs and KIIs lasted approximately one hour. At the end of data collection, the facilitators felt that saturation was achieved.

FGDs were conducted Runyankore. KIIs were conducted in either English or Runyankore, based on participant preference. All FGDs and KIIs were audio recorded. Field notes were recorded by a HCU employee (1 female) who accompanied the facilitators to FGDs and KIIs. Recordings were transcribed by an experienced local transcriber (1 female) who previously preformed similar duties for other Mbrara University of Science and Technology projects with good results. The transcriber listened to the recordings in Runyankore and directly translated into English. On two occasions, HCU staff checked the quality of transcription by selecting a transcript and re-listening to the audio recording while reading the transcribed text. On both occasions HCU staff were satisfied with the quality of transcription.

English transcripts were coded in Nvivo 9 and then analyzed by the main author, who was not involved with the study intervention. Thematic analysis strategies were used to code the data, which involved familiarization with data, identification of the emerging thematic framework, memos, and mapping [24]. Data were further conceptualized through the development of word frequencies, “clouds” and models. Emergent themes were tested through queries and classification of data into gender (male, female, mixed data), type of respondent (CHW, caregiver, health worker, government), and type of data (FGD, KII) from which additional queries were run. The FGD facilitators, who were also skilled in qualitative data analysis, cross-checked results and provided input.

Informed consent was obtained from all participants. Due to the low literacy level of CHWs and community members and some local leaders, the study was explained in detail by the facilitators before commencing the FGD or KII. The informed consent form was then read to the individual or group, and each participant was asked to either sign or provide a thumbprint at the bottom of the consent document to indicate his/her consent. Other literate participants were also verbally informed about the objectives of the study and then invited to read and sign the consent form

### Limitations

The purpose of this study was to contextualize a larger quantitative study and provide insights into stakeholders' perceptions of the iCCM project and its impact on access to health care. While every attempt was made to include respondents who represented the population in the study area, our ability to engage community members was limited by the need to have CHWs identify and invite participants to FGDs, which opens the opportunity for selection bias. Moreover, only community members who sought help from a CHW were included in FGDs. Opinions from community members who did not access CHW services are not included in this study. Due to study limitations it was not possible to share transcripts with participants and gather feedback. Results from this study cannot be generalized beyond the study group, although results are similar to other qualitative studies that examined perceptions of iCCM, most notably the study by Callagan-Koru [9].

## Results

### Participant demographics

A total of 20 data collection sessions were carried out (15 FGD and 5 KII) involving a total of 106 participants (54% female). At the end of data collection, saturation was achieved as facilitators noted that themes began to repeat and novel insights ceased to emerge. Table 3 shows the composition of respondents surveyed.

**Table 3:** Respondents who Participated in Post-Intervention Qualitative Study by Category and Gender.

Focus Group Discussions				
Stakeholder Group	# of FGDs	Participants		
		Male	Female	Total
Local Leaders	2	11	1	12
CHWs	6	13	34	47
Caregivers	6	19	16	35
Health Workers	1	1	6	7
**Total FGDs**	**15**	**44**	**57**	**101**

Key Informant Interviews				
Stakeholder Group	# of FGDs	Participants		
		Male	Female	Total
Sub-County Chairman	1	1	0	1
Sub-County Chief	1	1	0	1
District health Officer	1	1	0	1
Health, Health & Social Services	1	1	0	1
District council Leader	1	1	0	1
**Total Key Informant Interviews**	**5**	**0**	**0**	**5**

Main themes identified were: improved access to health services, trust in CHWs, CHWs as a link to formal health facilities, improved health outcomes, and economic benefits as a result of the iCCM CHW intervention.

### Improved access to health services

Caregivers reported improved access to health care for their young sick children as a result of the iCCM CHW intervention. Caregivers felt that access was improved because it was closer to home, available, and reliable. When a child was sick, care could be accessed at any time of the day or night, and care was received sooner because it was available closer to home. Besides being closer, access to iCCM CHWs was reported to be more reliable than services offered at formal health facilities; iCCM CHWs were almost always available, unlike health centres, which might be closed, without staff, or have medicine stock-outs. In cases of an iCCM CHW absence, many caregivers reported being able to visit another iCCM CHW, from the same or another village. Occasionally, respondents reported seeking care from a pharmacy or other public provider during an iCCM CHW absence. Overall, respondents expressed gratitude for the iCCM CHW services and requested that the program be continued.

*“When a child gets sick late in the night, you have [to] run to hospital to access treatment for the child. In calling for help from these nurses [at a health centre], the child could even die in your arms trying to call them but now with the [iCCM CHWs] in the village, treatment is accessed at any time. And also before there would be a long line at the health centre unlike with the [iCCM CHWs].”* [Caregiver, FGD]

*“My child suffers from pneumonia. She became badly ill one night and at around three am I had to move to the [iCCM CHW's] home as of course the hospitals were closed. So she welcomed me and treated the child with care until around six am, when we took the child to Kyabugimbi.”* [Caregiver, FGD]

One important access concern expressed by all respondents, though not directly related to the question of care for children under five years, related to the regulation that iCCM CHWs were authorized to treat only children under the age of five years, which is a limitation set by National Ugandan iCCM guidelines. ICCM CHWs, caregivers, and health centre staff all spoke about the under-five-years treatment restriction as a significant problem causing frustration and even conflict. Some respondents cited examples of caregivers sometimes lying about a child's age or sharing medicine prescribed for an eligible child in order to bypass the age limitation. In other cases, children older than five were taken to a traditional healer, pharmacy or other local provider.

*“ …A woman brought me a sick child and in my treatment I stop at the age of five so I couldn't handle the case. So I gave her a referral form for the child's treatment to take it to hospital. But when she reached home, they told her that the child had other diseases so they didn't take her to hospital but to the traditional healers.”* [CHW, FGD]

Caregivers who participated in this study expressed gratitude for the iCCM CHW program, and the resulting improved access to health care for their young children. Gratitude was discussed in terms of distance but also in terms of reliability, both of which contributed to an overwhelming request for the continuation of the iCCM CHW program.

### Communities trust iCCM CHWs to treat sick children

Caregivers told us they appreciate the work that iCCM CHWs do for their children and their communities using terms such as “*compassion*,” “*trust*,” “*care*” and “*welcome.*” Moreover, many respondents from all stakeholder groups recognized the great sacrifices made by volunteer iCCM CHWs to be available for their communities; iCCM CHWs were reported to leave their farms, wake in the middle of the night, and neglect their own families in order to help their communities. Our data indicate that these feelings of appreciation impact caregivers' willingness to utilize iCCM CHWs. The following quotations exemplify this appreciation for the care offered by iCCM CHWs.

*“When you take [to an iCCM CHW] a sick child, you see the way they care about the child and are doing their best to cure the child.”* [Caregiver, FGD]

*“The reason why I trust [the iCCM CHWs] is that some of us we stay deep in the mountains but when you call upon them, they are willing to come and treat the sick child.”* [Caregiver, FGD]

Importantly, many of our respondents from all stakeholder groups reported appreciation for the compassionate care offered by iCCM CHW, and the sacrifices sometimes necessitated by the role. Many respondents from all stakeholder groups requested that iCCM CHWs be compensated with “*at least a token*” for their work. Often this requested “token” was money, but other respondents suggested that the program provide iCCM CHWs with supplies, such as a bicycle, to make the role of iCCM CHWs easier. Other respondents suggested that a program be established to provide assistance to iCCM CHWs, such growing food or caring for children while CHW's performed their duties.

### CHWs link communities and health facilities

According to respondent input, the iCCM CHW intervention provided an important, and previously missing, link between health facilities and remote communities. Caregivers, local leaders and CHWs expressed this link primarily in terms of drugs that were available closer to families while health workers and government officials spoke about CHWs extending the reach of the public health system. While medicine distribution by iCCM CHWs was described as a valuable service, iCCM CHWs and health workers recognized that some illnesses were beyond the capacity of CHWs, who were trained to provide care for only uncomplicated fever, cough and fast breathing, and diarrhea.

*“I believe they [iCCM CHWs] are well trained because when they realize that they cannot handle the case with a sick child, they write a referral form and the child is taken to the hospital.“* [Health Centre Staff, FGD]

This iCCM CHW-health centre connection worked two ways. ICCM CHWs regularly connected with health centre staff to ask questions, make referrals, and collect medicines, while health centre staff appreciated knowing iCCM CHWs assessed children, referred difficult cases to a health facility, and provided a description of the problem. In some instances the iCCM CHW accompanied a sick child to a health facility.

Caregivers also appreciated the iCCM CHW-health facility linkage. Several respondents described arriving at a health centre with an iCCM CHW referral form and being welcomed and quickly served by health centre staff:

*“When you approach the [iCCM CHW] and she finds that she cannot handle the case, they have phones to communicate with nurses. Like, ”Nurse Barbra, I am sending you a sick child whom I have failed to handle.” So when you go with the referral form you don't wait long and they even care about you.”* [Caregiver, FGD]

Three caregivers in our study expressed concern about iCCM CHWs giving out medications in communities. One caregiver worried that uneducated iCCM CHWs might not know when medicines had expired; while another worried that the iCCM CHW might misdiagnose an illness. The third caregiver told a story about her child who was incorrectly treated by an iCCM CHW but then referred the child to a health facility, where the child was correctly diagnosed and treated. This informant said:

*“I took the child [to the iCCM CHW] who had cough and high fever. He gave me tablets that I gave to the child but [the child] did not become fine so I took back the child and [the iCCM CHW] referred me to a health centre. When I reached the health centre they found that the child had measles so the child was treated.”* [Caregiver, FGD]

While the majority of our data is very positive about the role of iCCM CHWs, our data also indicate that some caregivers were worried about uneducated and voluntary iCCM CHWs diagnosing illness and distributing medicines. However, during the course of this study, numerous iCCM CHWs in one area identified children whose illness did not fit within the treatment protocols provided to iCCM CHWs and referred these cases to health facilities. Several of these children were diagnosed with measles at the health facility, which alerted health workers to a measles outbreak and resulted in an immunization campaign. This event emphasizes the importance of having trained iCCM CHW's who are linked to health facilities present in communities.

### Improved health outcomes due to iCCM by CHW's

Community members, local leaders, and health workers unanimously thought the iCCM CHW intervention improved health outcomes for young children in intervention communities. Health officials who participated in this study reported an increase in women delivering in health facilities and higher attendance rates at immunization clinics in iCCM areas. Health centre staff reported a decrease in the number of children brought to health facilities for uncomplicated illness, an observation that was attributed to iCCM CHWs caring for children in communities. Caregivers reported that iCCM CHWs had “*saved*” their children, “*reduced the death rate*” of children and contributed to the “*disappearanc*e” of some children's diseases (most notably diarrhea) in their communities. Moreover, iCCM CHW's reported that medicines distributed by iCCM CHWs were effective and saved lives. One iCCM CHW commented:

*“When I think of the young children below five in our [village] it is that they [caregivers] no longer have to take them [children] to Kyabugimbi [health centre] because most diseases like cough and high fever are now being treated here by me.”* [CHW, FGD]

### Economic benefits due to improved access

Numerous respondents, from all participant groups, spoke about economic gains resulting from the iCCM CHW intervention. Interestingly, while few respondents mentioned saving money due to the reduced cost of medicine, most focused on money saved as a result of reduced transportation costs and time away from important income-generating activities (farming) and household work. In communities where the majority of people survive on subsistence agriculture, time spent away from farms can result in less agricultural output, reduced stores of food, and fewer family members to contribute labor, especially if the illness requires one or more caregivers to be absent for extended periods of time. One caregiver had this to say:

*“When the services reached our local cells, the women were happy to accept them as they have helped the children and have reduced transport costs.”* [Caregiver, FGD]

iCCM CHW respondents also noted economic benefits for community members who could now access free health services from within their communities:

*“There is appreciation [from the community] since they know that the medicines are available. In case of any problem with their children they can easily access us and go back to their business like digging [farming] unlike before where they would go to hospital and spend more time there.”* [CHW, FGD]

*“I feel good because I help people save their money. When I treat a child and [he/she] gets well and the money that would have been used for treatment is now used for other purposes like buying books.”* [CHW, FGD]

Study participants also reported indirect economic benefits of iCCM CHWs. One health official explained that, culturally, neighbours and extended family in Uganda often help each other in difficult times, which can become a hardship for all community members when a child falls ill:

*“Culturally here when a neighbour is sick, you can check on this person, give food. It may be a drink, or money. So there are some other costs you tend to save when your child or your neighbour's or someone else's child is healthier.* [Government Official, KII]

While our study did not directly measure economic savings, these qualitative data highlight community perceptions of the economic benefits of the iCCM CHW program.

## Discussion

Following a short (eight month) iCCM CHW intervention in rural Uganda, access to health services for sick young children was perceived to be improved in both “traditional” and “other” measures of health care access. Services were accessed closer to home, iCCM CHWs were more reliably available day and night compared with health providers at health centres, and CHWs were trusted to be caring in their roles. Importantly, better access was understood to result in better health outcomes for young children and reduced costs for families, especially reducing the costs of transportation and other costs associated with accessing health care from formal health facilities. These findings complement quantitative treatment record data collected during this study, which indicates that 93% of children treated by iCCM CHW's in intervention areas received treatment consistent with iCCM treatment algorithm recommendations (96%, 90% and 92% for fever, pneumonia and diarrhea respectively)[20]. While quantitative data indicates that children in intervention areas received accurate treatment for fever, pneumonia and diarrhea, our qualitative study indicates that families, iCCM CHWs, local leaders, district health officials and health centre staff thought that treatment was also more accessible, reliable and economically beneficial to families.

Quantitative measurements of “health service access” typically measure outcomes such as the number of patients treated, distance to a health facility, cost, and proportion of children receiving care within 24 hours. However, stakeholder impressions of “other” measures of access, such as perceptions of quality of care, perceived trustworthiness of the care provider, and the impact of care on patients and families, are also important in order for programs to be accepted by communities. Results from this study support research by Callaghan-Koru [9] that reported on health workers and managers in Malawi who perceived that iCCM delivered by CHWs contributed to increased geographic access to good quality health care, at all hours of the day, and reduced caseloads at health facilities. Results from this study, however, expand on Callaghan-Koru's [9] findings by examining “other” aspects of access. Caregivers in this study reported that they *felt* good about the services offered by CHWs. As health systems endeavor to provide high quality services that are delivered through sustainable programs, community perceptions of these services matter; individuals will not use services that they do not feel address their needs in a respectful or effective manner [15]. From our qualitative study, respondent stories about iCCM CHWs who willingly left their farms, woke during the night to assess a critically ill child, accompanied a mother and sick child to a health facility, and took time to follow up the day after treatment indicate that iCCM by CHW can improve health outcomes for young children and make health services more accessible for rural families.

While respondents in this study often spoke of “barriers” to health facilities, they also spoke of iCCM CHWs in terms of “access”. This access was discussed both in terms of commonly cited measures of access to health care such as shorter distance and less costly services, as well as other less commonly discussed measures of access such as health care that was perceived to be more caring and trustworthy. In this study, iCCM CHWs were frequently reported to have welcomed sick children into their homes, provided compassionate care, and offered quality treatment to sick children. Moreover, the iCCM CHW program was perceived to save time, money, and lives.

Interestingly, caregivers who first sought care from an iCCM CHW and were subsequently referred to a health facility reported kinder, more welcoming, and more timely care at the health facility due to the iCCM CHW referral. These findings emphasize the importance of linkages between iCCM CHWs and health facilities.

Strong stakeholder support for an iCCM by CHW intervention in a rural Ugandan community suggests that iCCM by CHW improves access to care, and that the care was perceived to be good. Moreover, stakeholders associated access to better care with better health outcomes, decreased cost, and stronger community-health facility linkages. This study supports the need for increased investment and momentum towards scale-up of iCCM by CHW, especially in very remote communities. However, issues raised related to community concerns around age cut-offs emphasize the importance of careful consideration of operational policies and communication around current Ugandan iCCM by CHW policy that restricts iCCM treatment to children under five years of age. Also, the finding that iCCM CHW programming seemingly improved impressions of care received at health facilities suggests that community members and health workers are open to working together towards a shared goal of better health; community-based iCCM by CHW should not supplant efforts to improve health facility capacity. More studies on the interactions of CHWs and health centre staff and on longer-term iCCM interventions would improve understanding and planning within iCCM programming.
